# Microstructure and Mechanical Properties of AlTiVCuN Coatings Prepared by Ion Source-Assisted Magnetron Sputtering

**DOI:** 10.3390/nano13243146

**Published:** 2023-12-15

**Authors:** Haijuan Mei, Kai Yan, Rui Wang, Lixia Cheng, Qiuguo Li, Zhenting Zhao, Ji Cheng Ding, Weiping Gong

**Affiliations:** 1 Guangdong Provincial Key Laboratory of Electronic Functional Materials and Devices, Huizhou University, Huizhou 516007, China; haijuanmei@hzu.edu.cn (H.M.); lixia02741@163.com (L.C.); liqiuguo10@163.com (Q.L.); zhzhting@hzu.edu.cn (Z.Z.); 2 Department of Materials Science and Engineering, Southern University of Science and Technology, Shenzhen 518000, China; 3 School of Mechanical Engineering, Guilin University of Aerospace Technology, Guilin 541004, China; wanrui@guat.edu.cn; 4 School of Materials Science and Engineering, Anhui University of Technology, Maanshan 243002, China; jcdingxinyang@126.com

**Keywords:** ALIS, ion source power, microstructure, mechanical properties

## Abstract

The AlTiVCuN coatings were deposited by magnetron sputtering with anode layer ion source (ALIS) assistance, and the microstructure and mechanical properties were significantly affected by the ion source power. With increasing the ion source power from 0 to 1.0 kW, the deposition rate decreased from 2.6 to 2.1 nm/min, and then gradually increased to 4.0 nm/min at 3.0 kW, and the surface roughness gradually decreased from 28.7 nm at 0 kW to 9.0 nm at 3.0 kW. Due to the enhanced ion bombardment effect, the microstructure of the coatings changed from a coarse into a dense columnar structure at 1.0 kW, and the grain size increased at higher ion source powers. All the coatings exhibited c-TiAlVN phase, and the preferred orientation changed from the (220) to the (111) plane at 3.0 kW. Due to the low Cu contents (1.0~3.1 at.%), the Cu atoms existed as an amorphous phase in the coatings. Due to the microstructure densification and high residual stress, the highest hardness of 32.4 GPa was achieved for the coating deposited at 1.0 kW.

## 1. Introduction

Due to their unique physical properties, the Ti-Al-V-N coatings have been widely investigated, such as high hardness, and high temperature self-lubricating effect. Due to the hardening effect of c-TiAlVN phase, the alloying of V further enhanced the hardness of TiAlN coatings [1,2]. Additionally, due to the lubricating effect of V_2_O_5_ and V_n_O_2n−1_ oxides, the tribological properties of the TiAlN coatings were significantly improved by adding V, especially at elevated temperatures [3,4]. At 700 °C, the friction coefficient of TiAlVN coatings sharply decreased to 0.27 [5]. However, due to the limited oxidation at medium and low temperatures, the friction coefficient was relatively high, and even reached as high as 0.7~0.8 at room temperature (RT) and 0.9~1.0 at 500 °C for the TiAlVN coatings [1,6,7]. In addition, the ductility of the TiVN coatings was decreased by adding V [8]. Similarly, the brittleness of the Ti-Al-V-N coatings was also increased by adding V, and then increased the flank wear [9]. As a soft metal, Cu has been added into the coatings to further improve the properties, such as enhanced hardness and toughness [10,11], and has reduced the friction coefficient [12]. For example, by adding metal Cu to the TiAlN [13] and TiAlVN [14] coatings, the toughness and friction coefficient were improved.

The comprehensive performance of the coatings can be improved not only through the design of composition and structure, but also through the optimization of deposition process. Magnetron sputtering (MS) is often used for the deposition of transition metal nitride coatings with smooth surfaces, but also causes a relatively low deposition rate [15]. To further improve the ionization ratio of the sputtered material, ion beam-assisted deposition (IBAD) has been proposed, such as Kaufman ion source [16], end-Hall ion source [17], and anode layer ion source (ALIS) [18]. In which, the anode layer ion source (ALIS) is widely used in industry to etch the substrate before deposition to improve the adhesion. For example, the DLC coatings were deposited by ALIS-assisted magnetron sputtering (MS) and found that the deposition rate and adhesion were enhanced [19]. By adjusting the ion source power, nanocrystalline γ-Al_2_O_3_ coatings were successfully deposited by ALIS-assisted bipolar pulse magnetron sputtering (BPMS) [20]. Similarly, the BN [21] and CN_X_ [22] coatings were also prepared by ALIS assisted radio frequency magnetron sputtering (RFMS), both the deposition rate and hardness were improved with increasing the ion source power. In addition, the electrochemical properties were enhanced for the TiN coatings deposited by ALIS-assisted magnetron sputtering (MS) [23]. Thus, with the assistance of ALIS, the comprehensive performance of AlTiVCuN coatings can be further improved, and it is expected to increase the cutting life of coated tools in the field of high-speed machining.

However, there are few studies that report on the multi-component coatings prepared by ALIS-assisted magnetron sputtering (MS). In this work, to improve the gas ionization ratio, the AlTiVCuN multi-component coatings were deposited by magnetron sputtering with ALIS assistance at different ion source powers. Moreover, the relationship between microstructure evolution and mechanical properties has been explored systematically.

## 2. Experimental Details

### 2.1. Coating Deposition

**Figure 1:**
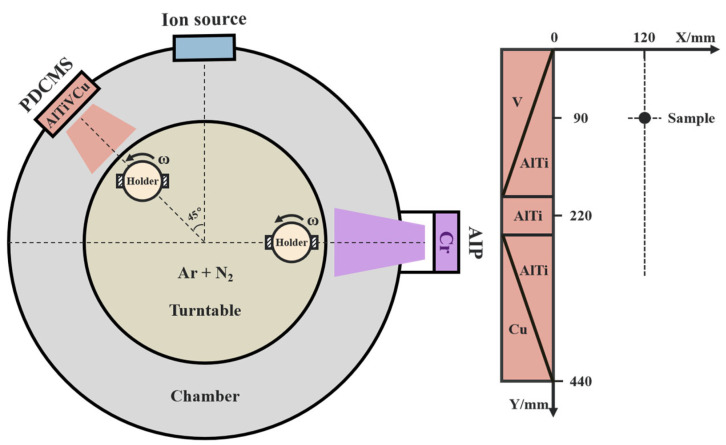
Schematic diagram of deposition system and sample position.

The AlTiVCuN coatings were prepared on the polished substrates by anode layer ion source (ALIS)-assisted pulsed dc magnetron sputtering (PMS) using Al_67_Ti_33_-V-Cu spliced target (69 × 443 mm, 99.9% purity). To increase the gas ionization ratio, ALIS was installed near the sputtered target, as Figure 1 shows. To achieve low Cu contents, the vertical distance of the sample position was set at 90 mm. The polished substrates, including the stainless steel and cemented carbide, were ultrasonically cleaned in the acetone and methanol for 20 min, respectively. Prior to deposition, the dried substrates were placed into the chamber, and then heated to 200 °C. To improve the adhesion strength between the coating and substrate, arc ion plating (AIP) equipped with Cr target (Ø100 × 20 mm, 99.99% purity) was first used to prepare a thin sub-layer of CrN (~320 nm) in a pure N_2_ atmosphere for 10 min, and the target current was kept at 100 A. Then, the AlTiVCuN coatings were prepared by using magnetron sputtering with ALIS assistance for 180 min. During the deposition process, the target power was controlled at 1.5 kW. As the only variable, different ion source powers (0, 1.0, 2.0, 3.0 kW) were used. Table 1 shows the other deposition parameters.

### 2.2. Coating Characterization

The chemical composition, morphology, and coating thickness were characterized by scanning electron microscopy (SEM, Nano430, Amsterdam, The Netherlands) equipped with EDS. An atomic force microscopy (AFM, Dimension Icon) was applied to measure the surface roughness in a tapping mode and operated at a scanning area of 15 × 15 μm^2^. An X-ray diffraction (XRD, Bruker D8 advance, Karlsruhe, Germany) was applied to characterize the crystal structure by using Cu *Kα* radiation (40 kV, 40 mA), operating in a scanning angle of 30~80° and a scanning step of 0.02°. The preferable orientation was determined by using the texture coefficient formula:

(1) $$T_{\left(hkl\right)}\;=\;\frac{I_{\left(hkl\right)}/I_{0(hkl)}\;}{\frac{1}{n}\sum_{n=1}^{n}I_{\left(hkl\right)}/I_{0(hkl)}}$$

where *I*_0(*hkl*)_ and *I*_(*hkl*)_, respectively, refer to the relative standard intensity of the TiN powder and intensity of the measured (*hkl*) peak, and n refers to the reflection number. X-ray photoelectron spectroscopy (XPS, Escalab 250Xi, Waltham, MA, USA) was used to identify the chemical structure by using Al *Kα* radiation. The microstructure was analyzed by transmission electron microscopy (TEM, Talos F200X, Thermo, Waltham, MA, USA) at an accelerating voltage of 200 kV. The TEM cross-section sample was prepared by focused ion beam (FIB, Auriga Carl Zeiss, Jena, Germany) using Ga ions. Based on Stoney’s equation and substrate curvature method, a film stress tester (FST-1000, Supro Instruments, Shenzhen, China) was applied to measure the residual stress of the coatings deposited on the stainless steel substrates. The hardness and elastic modulus were tested by nanoindentation (NHT^2^, CSM, Peseux, Switzerland). The maximum indentation load was set at 5.0 mN. At least five effective measurements were tested for each sample. The adhesion strength was measured by revetest scratch tester (RST, CSM, Peseux, Switzerland). The maximum scratch length and load were set at 3 mm and 120 N, respectively.

## 3. Results

### 3.1. Microstructure

**Figure 2:**
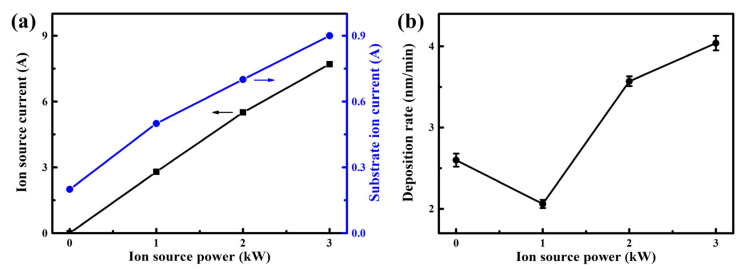
Ion source discharge current and substrate ion current (**a**), and deposition rate of the AlTiVCuN coatings (**b**) at various ion source powers.

Figure 2a shows the ion source current and substrate ion current of the AlTiVCuN coatings. With increasing the ion source power, the ion source current almost linearly increased from 0 to 7.7 A, which would contribute to an increase in the ion flux density [23]. Correspondingly, the substrate ion current also gradually increased from 0.2 to 0.9 A, indicating that the ion flux density delivered into the substrate surfaces increased, which would enhance the ion bombardment effect and the mobility of surface atoms. In Figure 2b, with increasing the ion source power, the deposition rate first decreased from 2.6 to 2.1 nm/min, and then gradually increased to 4.0 nm/min. The initial decrease in the deposition rate can be related to the enhanced ion bombardment effect. Generally, the coatings would become more compact under the bombardment effect of high-energy ions. In addition, the strong ion bombardment would cause a typical re-sputtering effect. At higher ion source powers, the gas ionization rate increased. After a series of collisions between the sputtered atoms and gas ions, the plasma density in the chamber increased, and more ions flew toward the growing coating surfaces under substrate bias voltage, contributing to an increase in the deposition rate. Similarly, the deposition rate of the BN [21] and α-CN_X_ [22] coatings also increased at higher ion source powers.

Table 2 lists the chemical composition of AlTiVCuN coatings at various ion source powers. As the ion source power increased from 0 to 1.0 kW, both the Al, Ti, V, and Cu contents decreased, whereas the N content increased from 53.6 to 62.9 at.%. With ALIS assistance, the gas ionization rate sharply increased and enhanced the activity of gas molecules and ion energy, resulting in more and more nitrogen ions delivered into the growing coatings [22,24]. At higher ion source powers, the ion flux density further increased, and more ions with high energy bombarded the substrate surface, which would cause a typical re-sputtering effect, especially for the lightweight element N. Thus, the N content in the coatings gradually decreased to 49.0 at.% at 3.0 kW, and the N/(Al + Ti + V) atomic ratios gradually decreased from 1.74 to 1.02, nearing to the stoichiometric ratio. When compared to titanium, aluminum has a lower relative atomic mass, which would be preferentially re-sputtered induced by the strong ion bombardment. Thus, the Al/Ti atomic ratios gradually decreased from 1.99 to 1.82 with increasing ion source power.

**Figure 3:**
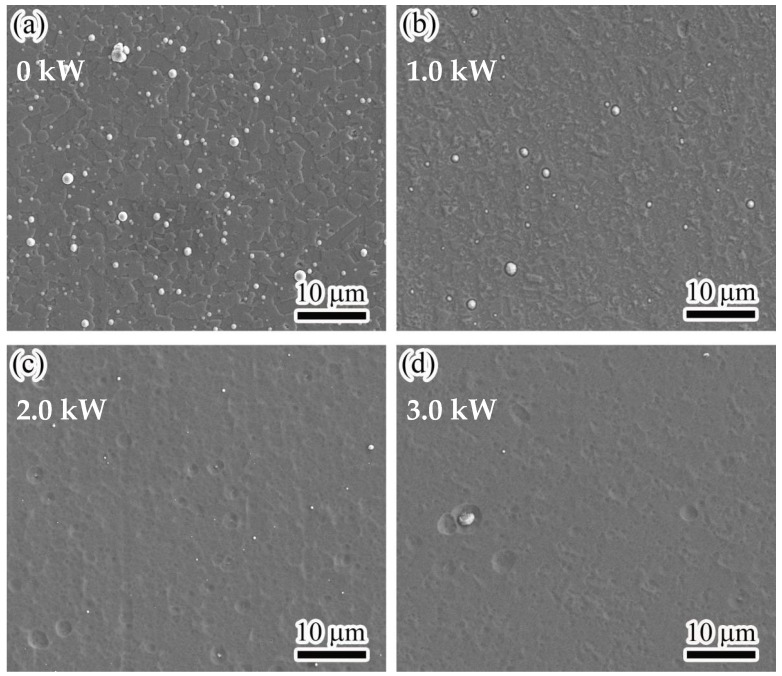
Surface micrographs of the AlTiVCuN/CrN coatings at various ion source powers: (**a**) 0 kW, (**b**) 1.0 kW, (**c**) 2.0 kW, (**d**) 3.0 kW.

**Figure 4:**
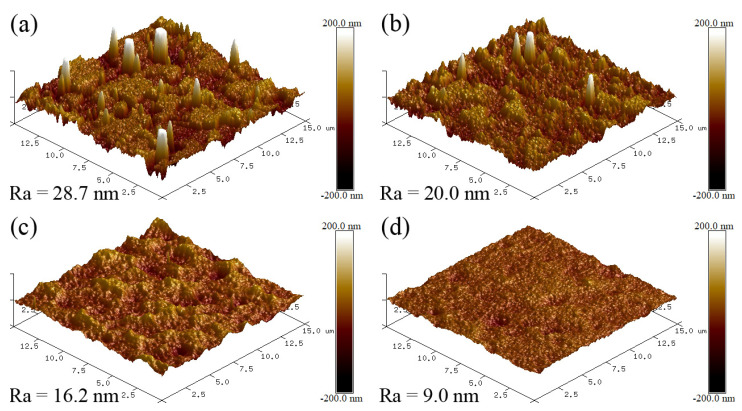
Surface roughness of the AlTiVCuN/CrN coatings at various ion source powers: (**a**) 0 kW, (**b**) 1.0 kW, (**c**) 2.0 kW, (**d**) 3.0 kW.

Figure 3 shows the surface micrographs of the AlTiVCuN/CrN coatings at various ion source powers. In Figure 3a, without the assistance of ALIS, the coating exhibited a plate-like structure and was accompanied by many microparticles, which could be related to the metal droplets generated during the deposition of the CrN sub-layer by AIP [25]. After applying ALIS, the gas ionization rate increased and then the ion bombardment effect was enhanced. At 1.0 kW, the plate-like structure became smaller and was accompanied by fewer and smaller microparticles, implying a decrease in the surface roughness. At high ion source powers above 2.0 kW, the plate-like structure almost disappeared, and the coating surfaces became much smoother, accompanied by some shallow pits and few microparticles. Due to the enhanced ion bombardment effect and etching effect at higher ion source powers, some weakly bound particles were bombarded out and then formed some shallow pits on the coating surfaces [21]. In addition, the mobility of surface atoms was also enhanced after ions collisions, which improved the smoothness of the coating surfaces. Thus, with increasing the ion source power, the coating surfaces changed from a plate-like structure to a shallow pit structure and became smoother due to the increased mobility of surface atoms. In Figure 4, the surface roughness decreased from 28.7 to 9.0 nm, which can be related to the structure evolution of the coating surfaces.

**Figure 5:**
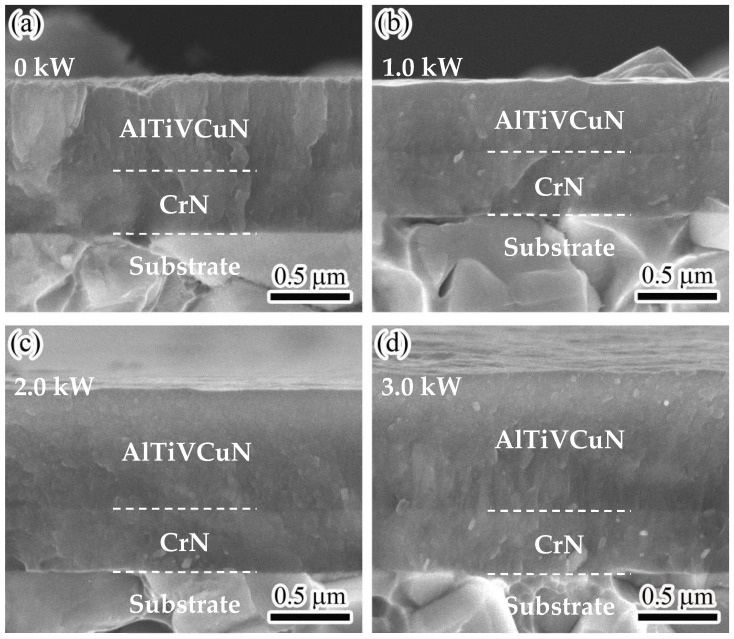
Cross-sectional micrographs of AlTiVCuN/CrN coatings at various ion source powers: (**a**) 0 kW, (**b**) 1.0 kW, (**c**) 2.0 kW, (**d**) 3.0 kW.

Figure 5 shows the cross-section images of the AlTiVCuN/CrN coatings at various ion source powers. An obvious double-layer formed in the cross-sections, in which a thin and dense sub-layer of CrN (~320 nm) was deposited by AIP. In Figure 5a, without ALIS assistance, a coarse columnar crystal morphology formed in the AlTiVCuN layer. At 1.0 kW, the coating changed into a dense and fine columnar crystal structure, which could be related to the enhanced ion bombardment with ALIS assistance [23]. A similar microstructure evolution was also observed in the Cr-Cu-N coatings under the ion beam bombardment with high energy [26]. With increasing ion source power, the thickness of AlTiVCuN coatings gradually increased, and the columnar grains became more obvious, especially for the coating deposited at a high ion source power of 3.0 kW as Figure 5d shows. At higher ion source powers, the gas ionization rate increased; after a series of ion collisions and energy transfer, more ions with high energy were delivered into the growing coating surfaces, which promoted grain growth and nucleation in the coatings. Additionally, the thermal effect induced by the strong ion bombardment also promoted slight grain growth [27].

**Figure 6:**
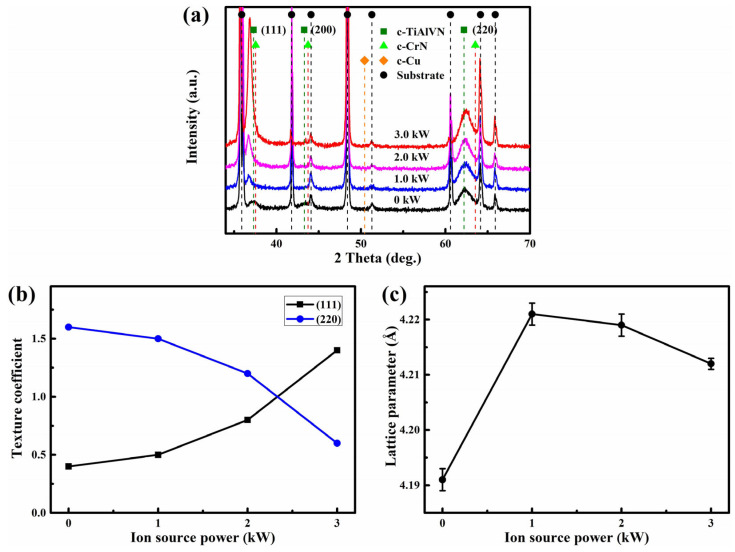
XRD pattern (**a**), texture coefficient (**b**), lattice parameter (**c**) of AlTiVCuN/CrN coatings at various ion source powers.

Figure 6a displays the XRD pattern of the AlTiVCuN/CrN coatings at various ion source powers. Three diffraction peaks appeared at around 37.3°, 43.3°, and 62.4°, which can be corresponded to the (111), (200), and (220) planes of the Ti-Al-V-N and Cr-N phase, respectively [3,9]. Due to the similar diffraction peak positions of the two face-centered cubic phases, the diffraction peaks of the sub-layer Cr-N were gradually covered by that of the top layer of Ti-Al-V-N. In addition to the diffraction peaks of nitrides, some strong and sharp diffraction peaks were also observed, corresponding to the diffraction peaks of the WC-Co substrate. However, due to the low Cu contents (≤3.1 at.%), no Cu-related phase appeared. A similar result was also observed in the Cr-Cu-N [26] coatings with low Cu contents. With increasing the ion source power, the weak (200) peak disappeared and the diffraction intensity of the (111) peak gradually increased. In Figure 6b, the evaluation of the preferred orientation was conducted by the texture coefficient *T*_(*hkl*)_ using Equation (1). At low ion source powers, the coatings presented a strong (220) preferred orientation with a high texture coefficient of 1.2~1.6. Due to the anisotropy of collision, the (220) orientation has a higher survival probability than that of the (111) orientation [25,28]. At 3.0 kW, the texture coefficient of the (111) plane gradually increased to 1.4, exhibiting a strong preferred orientation of the (111) plane. According to the thermodynamic model [29], when the strain energy dominated, the (111) preferred orientation would occur to minimize the total energy. A similar result was also found for the TiN coatings that strong ion bombardment favored the formation of the (111) preferred orientation [30]. In Figure 6c, with increasing the ion source power from 0 to 1.0 kW, the lattice parameter initially increased from 4.19 to 4.22 Å, and then gradually decreased to 4.21 Å at 3.0 kW, which can be related to the variation of residual stress.

**Figure 7:**
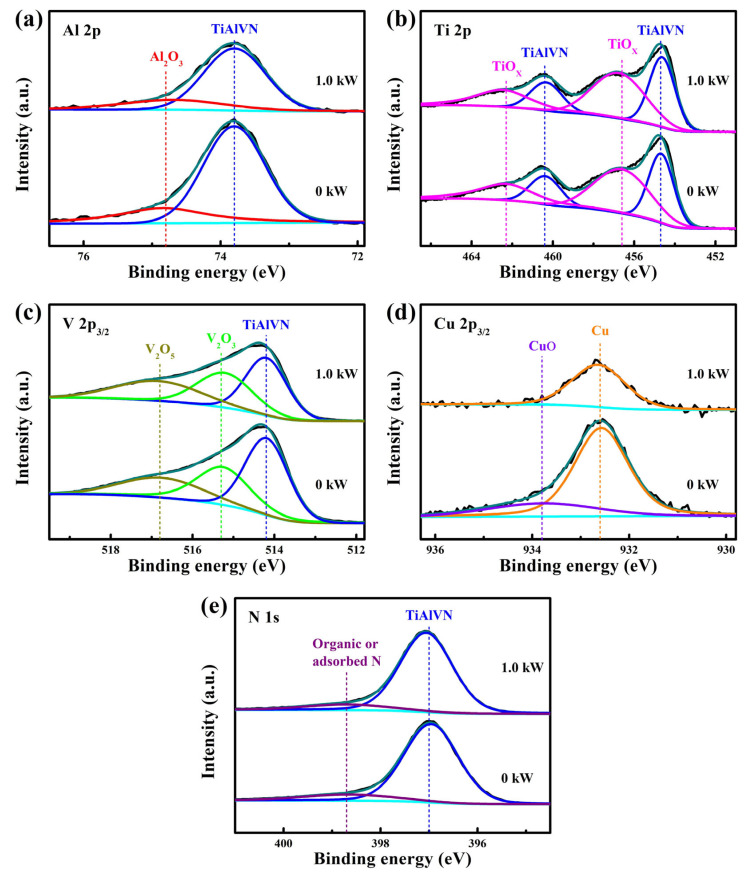
Fitted XPS spectra of the AlTiVCuN coatings: (**a**) Al 2p, (**b**) Ti 2p, (**c**) V 2p_3/2_, (**d**) Cu 2p_3/2_, and (**e**) N 1s.

To study the chemical bonding state of Cu, the chemical structure was analyzed by XPS. As Figure 7a shows, two peaks at 73.8 and 74.8 eV appeared in the Al 2p spectra, corresponding to the formation of TiAlVN and Al_2_O_3_, respectively [25]. In Figure 7b, the asymmetric Ti 2p spectra is deconvoluted into two group peaks, corresponding to the nitride peaks of TiAlVN (454.7 and 460.4 eV) and the minor oxide peaks of TiO_X_ (456.6 and 462.3 eV) [31]. The existence of oxide peaks would be due to the slightly oxidation of the coating surfaces that induced by the absorbed oxygen [32]. In Figure 7c, the V 2p_3/2_ spectra are deconvoluted into three peaks at 514.2, 515.3, and 516.8 eV, which could be assigned to the TiAlVN, V_2_O_3_, and V_2_O_5_, respectively. In Figure 7d, two peaks appear in the Cu 2p_3/2_ spectra, including the Cu peak at 932.6 eV and the CuO peak at 933.8 eV, implying that the Cu existed in the form of pure metallic [26]. In Figure 7e, the N 1s spectra is also deconvoluted into two peaks at 397.0 and 398.7 eV, which can be assigned to the TiAlVN and the organic or adsorbed N, respectively. With increasing the ion source power, the peak areas of the Al 2p, V 2p_3/2_, and Cu 2p_3/2_ spectra decrease are obvious, which can be related to the decrease in element contents as Table 2 shows.

**Figure 8:**
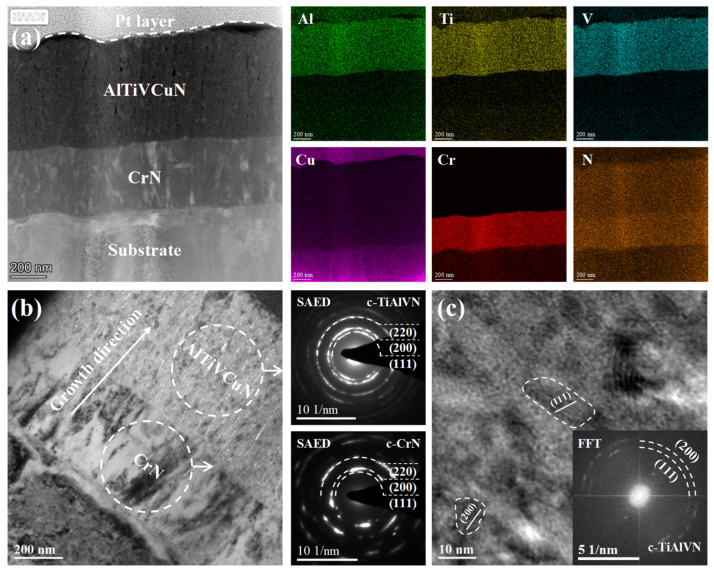
Cross-sectional TEM images of the AlTiVCuN/CrN coating deposited at 2.0 kW: (**a**) HAADF image and STEM mapping, (**b**) bright-field image and SAED pattern, (**c**) high-resolution image and FFT pattern.

Figure 8 shows the cross-sectional images of the AlTiVCuN/CrN coating deposited at 2.0 kW. From the HAADF image in Figure 8a, a double-layer structure can be clearly seen, including the CrN and AlTiVCuN layer. Based on the STEM mapping, the coating elements, including the sub-layer of Cr and N, and the top layer of Al, Ti, V, Cu, and N, were distributed uniformly in the cross-section. However, due to the low Cu contents detected by EDS (Table 2), the elemental mapping of Cu was not very clear, but the existence of Cu has been demonstrated by XPS. In addition, a clear multi-layer interface can be identified, indicating that the element diffusion did not occur inside the coatings during the deposition process. In Figure 8b, it can be seen that some coarse columnar crystals formed in the CrN sub-layer, exhibiting a typical columnar crystal structure. From the SAED pattern, the incontinuous diffraction spots can be assigned to the (111), (200), and (220) planes of the CrN phase. As for the top layer of AlTiVCuN, a fine and incontinuous columnar structure formed along the growth direction in the cross-section. The SAED pattern revealed fine and continuous diffraction rings, indicating that much smaller crystalline grains formed in the top layer deposited by ALIS-assisted magnetron sputtering. These continuous diffraction rings can be assigned to the TiAlVN phase. In addition, due to the low Cu content, no Cu-related phase can be found, demonstrating that the Cu atoms existed as an amorphous phase in the coatings. A similar amorphous structure of Cu was also found in the Mo-Cu-N [12] and TiAlN/Cu [33] coatings at low Cu contents. In Figure 8c, from the high-resolution image and corresponded FFT pattern, two lattice planes with d-spacing of 0.2433 and 0.2110 nm can be seen, corresponding to the c-TiAlVN phase.

### 3.2. Mechanical Properties

**Figure 9:**
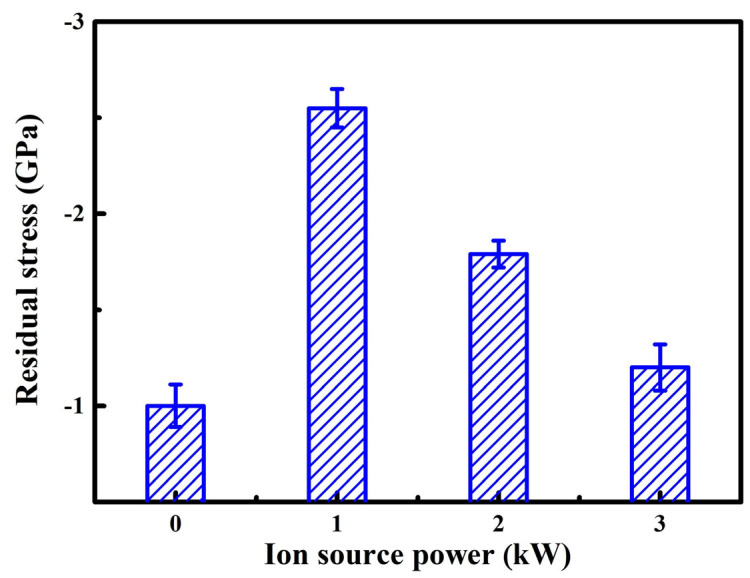
Residual stress of the AlTiVCuN/CrN coatings at various ion source powers.

Generally, the residual stress often includes the thermal stress and intrinsic stress in the coatings [34]. Compared to the 316 L stainless steel substrate, the AlTiVCuN and CrN coatings have a lower coefficient of thermal expansion, resulting in a typical compressive thermal stress in the nitride coatings; whereas the intrinsic stress is often caused by the deposition conditions, which often causes a relatively high compressive intrinsic stress. Based on the Stoney’s equation and substrate curvature method, the residual stress of the AlTiVCuN/CrN muti-layer coatings can be measured as Figure 9 shows. All the coatings exhibited negative values, indicating the formation of compressive residual stress. Without ALIS assistance, the coating showed a low compressive residual stress of 1.0 GPa. After applying ALIS, the gas ionization rate increased and enhanced the ion bombardment effect. At 1.0 kW, the compressive residual stress sharply increased to 2.6 GPa. Thus, the initial increase in the compressive residual stress can be related to the enhanced ion bombardment effect [35]. However, with further increasing the ion source power, the compressive residual stress gradually decreased to 1.2 GPa. At higher ion source powers, the partial relaxation of residual stress can be related to the increase in coating thickness. For the thinner coatings, the residual stress decreased with increasing the thickness [36]. In addition, the decrease in residual stress can be related to the texture change from the (220) to the (111) orientation. A similar result was also found for the TiN coatings where the residual stress decreased with developing the (111) texture [37].

**Figure 10:**
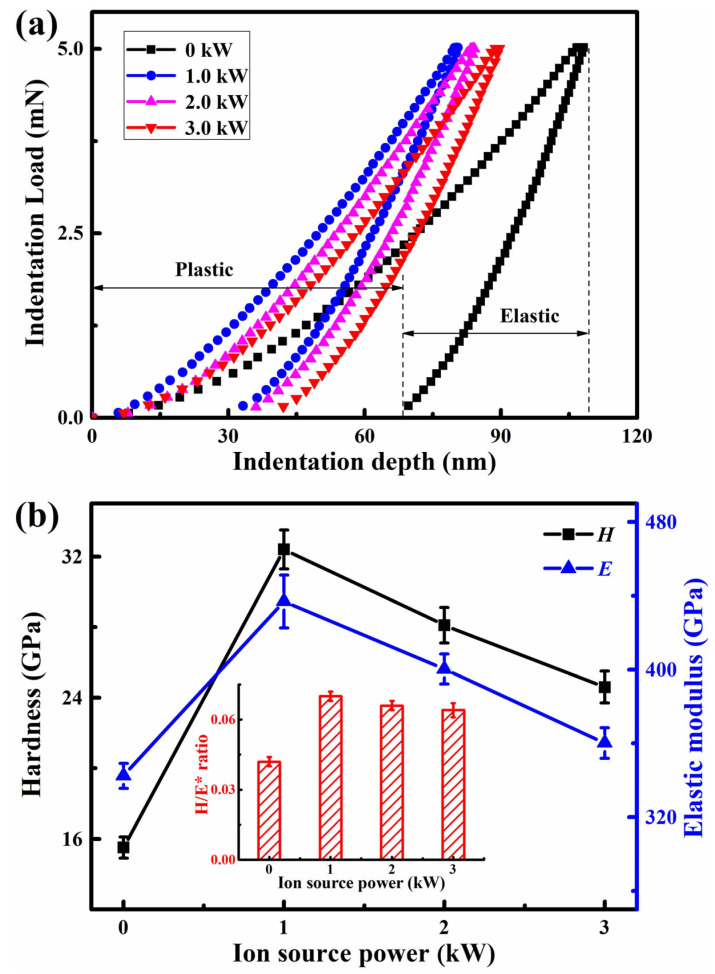
Indentation load–displacement curves (**a**), hardness, elastic modulus, and H/E* ratio (**b**) of the AlTiVCuN/CrN coatings at various ion source powers.

Figure 10a shows the indentation load–displacement curves at a maximum load of 5 mN. To reduce the effect of cemented carbide substrate, it can be seen that the maximum indentation depth kept less than 10~15% of the total thickness of the AlTiVCuN/CrN coatings. Due to the thin thickness of the AlTiVCuN coatings (Table 2), the measured hardness belonged to the comprehensive hardness of multi-layer coatings. Based on the indentation load–displacement curves, the hardness and elastic modulus of the coatings can be achieved. In Figure 10b, without ALIS assistance, the coating shows a relatively low hardness of 15.5 GPa and elastic modulus of 342.4 GPa, which can be related to the coarse columnar structure and low compressive residual stress. After applying ALIS, the ion bombardment was enhanced, and the highest hardness of 32.4 GPa was achieved for the coating deposited at 1.0 kW. This can be explained by the microstructure densification and the sharp increase in compressive residual stress [21]. At higher ion source powers, the residual stress gradually decreased, as Figure 9 shows, leading to a decrease in the hardness and elastic modulus. A similar result was also found for the TiN coatings where the hardness decreased when the ALIS discharge current increased above 3.5 A [23]. In addition, the decrease in the coating hardness was also related to the increased grain size as Figure 5 shows. It has been reported that the enhanced hardness of BN coatings can be attributed to the decrease in grain size [38]. Thus, the hardness can be affected by various mechanisms, including the microstructure evolution, residual stress, and grain size. With increasing ion source power, the H/E* ratio initially increased and then decreased, and the highest H/E* ratio of 0.07 was achieved at 1.0 kW, implying that the best elastic deformation ability was achieved for the coating.

**Figure 11:**
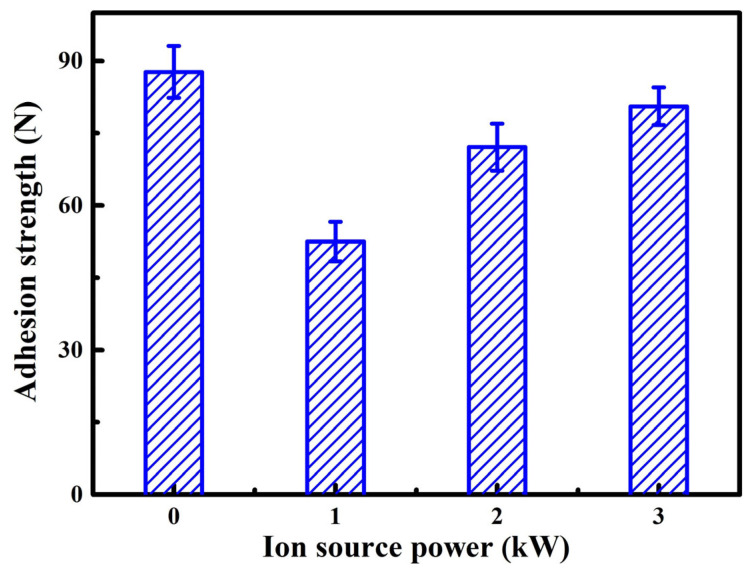
Adhesion strength of the AlTiVCuN/CrN coatings at various ion source powers.

Figure 11 presents the adhesion strength of the AlTiVCuN/CrN coatings at various ion source powers. During the scratching test, the failure mode L_C2_, at which the adhesive chipping occurred at track edges and exposed the substrate, is often used to determine the adhesion strength. Thus, the measured adhesion strength belonged to the bonding force between the multi-layer coatings and the substrates. With increasing the ion source power, the adhesion strength first decreased from 87.7 to 52.5 N, and then gradually increased to 80.6 N. The variation of adhesion strength could be affected by many factors, including the interface bonding, substrate hardness, coating thickness, and properties. Among these, the residual stress has a significant impact on the adhesion strength of the coatings. Due to its compressive nature, a relatively high compressive residual stress often causes a low adhesion strength [25]. The first decrease in the adhesion strength can be explained by the sharp increase in residual stress in the coatings. At higher ion source powers, the assisted energy delivered into the growing coatings increased, which would modify the interface between the top-layer and sub-layer coatings. Similarly, for the TiN coatings, with ALIS assistance, the adhesion strength was increased due to the enhanced interface bonding [23]. In addition, the relaxation of residual stress also contributed to the improvement of adhesion strength at higher ion source powers.

## 4. Discussion

In this study, the AlTiVCuN coatings were deposited by magnetron sputtering with ALIS assistance. In order to further improve the adhesion strength between the coating and substrate, a thin CrN layer (~320 nm) was first deposited by arc ion plating (AIP) for 10 min, and then formed a multi-layer structure of AlTiVCuN/CrN. The introduction of the CrN layer has a certain impact on the microstructure and mechanical properties of the coatings. Firstly, during the deposition of the CrN layer by AIP, many large metal droplets were easily generated, which flew toward the substrate surface and then formed large microparticles on the coating surface, resulting in an increase in the surface roughness. Secondly, from the cross-sectional TEM image of the coating, it can be seen that the CrN layer exhibited a typical face-centered cubic structure with the (111), (200), and (220) planes, which was beneficial for promoting the nucleation and growth of the AlTiVCuN top layer and formed a similar face-centered cubic structure. Finally, the mechanical properties, including the residual stress, hardness, and adhesion strength, corresponded to the comprehensive properties of the AlTiVCuN/CrN multi-layer coatings. Due to a good interface bonding which formed between the sub-layer of CrN and the top-layer of AlTiVCuN, all the coatings exhibited a high adhesion strength that ranged from 52.5 to 87.7 N.

Without the assistance of ALIS, the AlTiVCuN coating showed a coarse columnar crystal structure and a low deposition rate of 2.6 nm/min and resulted in a relatively low hardness of 15.5 GPa and elastic modulus of 342.4 GPa, which can be related to the low gas ionization ratio. After applying ALIS, both the ion source current and substrate ion current almost linearly increased with increasing the ion source power, which would enhance the ion bombardment effect and the mobility of surface atoms, resulting in a gradual decrease in the surface roughness from 28.7 to 9.0 nm. At 1.0 kW, the coatings changed into a dense and fine columnar crystal structure, contributing to the highest hardness of 32.4 GPa. With further increasing the ion source power, the coating hardness gradually decreased, which was mainly related to the relaxation of compressive residual stress and the increase in grain size in the coatings.

## 5. Conclusions

In this study, the ion source power has a significant effect on the microstructure and mechanical properties of the AlTiVCuN coatings deposited by ALIS-assisted magnetron sputtering. With increasing the ion source power from 0 to 1.0 kW, the deposition rate decreased from 2.6 to 2.1 nm/min and then gradually increased to 4.0 nm/min at 3.0 kW. Without ALIS assistance, the coating exhibited a coarse columnar crystal structure. After applying ALIS, the gas ionization rate increased and then the ion bombardment effect was enhanced. The coatings changed into a dense and fine columnar crystal structure at 1.0 kW and the surface roughness gradually decreased. At low ion source powers, the coatings showed a solution phase of TiAlVN with the (220) preferred orientation, and then changed into (111) preferred orientation at 3.0 kW. Due to microstructure densification and the increase in residual stress, the highest hardness of 32.4 GPa was achieved at 1.0 kW. With increasing the ion source power, the adhesion strength decreased from 87.7 to 52.5 N at 1.0 kW and then gradually increased to 80.6 N at 3.0 kW.

## Figures and Tables

**Table 1:** Deposition parameters of CrN sub-layer and AlTiVCuN layer.

Parameters	CrN	AlTiVCuN
Base pressure (Pa)	5.0 × 10^−3^	
Working temperature (°C)	200	
Working pressure (Pa)	0.5	0.9
Ar/N_2_ flow rate (sccm)	0/140	75/20
Substrate bias voltage (V)	–120	–150
Target to substrate distance (mm)	200	120
Target current (A)	100	
Target power (kW)		1.5
Duty cycle (%)		50
Ion source power (kW)		0, 1.0, 2.0, 3.0
Deposition time (min)	10	180

**Table 2:** Chemical composition and thickness of AlTiVCuN coatings analyzed by EDS and SEM.

Power (kW)	Chemical Composition (at.%)					Al/Ti Ratio	N/(Al + Ti + V) Ratio	Thickness (nm)
	Al	Ti	V	Cu	N			
0	18.1	9.1	16.1	3.1	53.6	1.99	1.24	468 ± 14
1.0	15.0	7.7	13.4	1.0	62.9	1.95	1.74	370 ± 9
2.0	20.5	11.0	10.4	1.6	56.5	1.86	1.35	642 ± 11
3.0	22.4	12.3	13.3	3.0	49.0	1.82	1.02	727 ± 17

## Data Availability

Data are contained within the article.
